# Characterization of Ethanol Extracted Cell Wall Components of *Mycobacterium avium* Subsp. *paratuberculosis*

**DOI:** 10.3390/vetsci6040088

**Published:** 2019-10-31

**Authors:** John P. Bannantine, Ashutosh Wadhwa, Judith R. Stabel, Shigetoshi Eda

**Affiliations:** 1 National Animal Disease Center, USDA-Agricultural Research Service, Ames, IA 50010, USA; judy.stabel@usda.gov; 2 Department of Forestry, Wildlife and Fisheries, University of Tennessee, Knoxville, TN 37996, USA; ashutoshwadhwa@gmail.com (A.W.); seda@utk.edu (S.E.)

**Keywords:** Johne’s disease, paratuberculosis, ELISA, lipid, antigens

## Abstract

Antigens extracted using ethanol (EtOH) and incorporated in the EtOH vortex ELISA (EVELISA) test have previously shown high specificity and sensitivity for detecting *Mycobacterium avium* subspecies *paratuberculosis* (*Map*) and *M. bovis* infections in cattle. The objective of this study is to define the components present in the EtOH extract. We show that this extract is composed of lipid, carbohydrate, and proteins on the surface of the bacilli, and that EtOH removes the outer layer structure of *Map* which comprise these elements. To identify proteins, polyclonal antibodies to the EtOH prep were produced and used to screen a *Map* genomic expression library. Seven overlapping clones were identified with a single open reading frame, MAP_0585, common to all. MAP_0585, which encodes a hypothetical protein, was recombinantly produced and used to demonstrate strong reactivity in sera from hyperimmunized rabbits, but this protein is not strongly immunogenic in cattle with Johne’s disease. A panel of monoclonal antibodies was used to determine the presence of additional proteins in the EtOH extract. These antibodies demonstrated that a well-known antigen, termed MPB83, is present in *M. bovis* EtOH extracts and a fatty acid desaturase (MAP_2698c) is present in *Map* EtOH extracts, while lipoarabinomannan was common to both. The lipid and carbohydrate components of the extract were analyzed using thin layer chromatography and lectin binding, respectively. Lectin biding and protease treatment of the EtOH extract suggest the antigenic component is carbohydrate and not protein. These results give further insight into this important antigen prep for detecting mycobacterial diseases of cattle.

## 1. Introduction

The ethanol (EtOH) extract of *M. avium* subspecies *paratuberculosis* (*Map*) is an extraction of highly antigenic cell wall components that consists of proteins, carbohydrates, and lipids. This antigen extract differs from the sonicated whole-cell extract of *Map* used in many research studies, and it has been useful in detection of Johne’s disease (JD) in dairy cattle. The idea to produce this extract was first had in 2005 when Eda et al. [1] used flow cytometry to demonstrate that antibodies in sera of *Map*-infected cattle bound to *Map* bacilli but not to other mycobacterial species. This observation led to the hypothesis that *Map* has unique antigens on its outer surface. Furthermore, the antibody-*Map* binding complexes were detected in natural bovine infections several months earlier than the fecal culture test or commercial ELISA test. The empirical diagnostic sensitivity and specificity of this novel flow cytometric assay was estimated to be 95.2% and 96.7%, respectively. These data suggested that by detecting antibodies in the cell wall of *Map* one could develop a diagnostic test to detect early *Map* infection, which included animals shedding low and medium amounts of bacteria in their feces. Therefore, the objective was to capture surface antigens while avoiding internal (cytoplasmic) antigens, which increased nonspecific reactivity of the ELISA test [2].

After testing a number of alcohols and other organic solvents at various concentrations on *Map*, our group found that an 80% EtOH solution, followed by a brief vortex agitation, was the most effective method for extracting antigens that specifically reacted with sera of *Map*-infected cattle [3]. An in-house ELISA was developed using the EtOH-extracted preparation and named EtOH vortex ELISA (EVELISA). This test showed an improvement over the flow cytometric assay with an empirical diagnostic sensitivity and specificity of 100% and 96.9%, respectively. Furthermore, the antigen-coated ELISA plates had a stable shelf life of at least 7 weeks. When the EVELISA test was adapted for use in detecting *M. bovis*-infected cattle, it also showed high diagnostic sensitivity and specificity for cattle and white-tailed deer [4]. The test has also been applied to farmed red deer [5], and can be used to test both serum and milk samples [6]. This 80% EtOH extraction method was used to produce the antigen throughout this study.

Despite the diagnostic value of this antigen preparation, little is known about its components and it has never been demonstrated that the EtOH extract yields mostly surface antigens. Many of the antigenic lipids of *Map* contain a carbohydrate component (i.e. the phenolic glycolipids, trehalose dimycolate, and lipooligosaccharides), while other lipids are associated with peptides comprising 3 or 5 amino acids [7,8,9]. The antigenicity of selected *Map* lipids, whether complexed with a carbohydrate moiety or peptide, has been a matter of dispute. For example, the well-studied Para-LP-01 lipid, also known as L5P, has been shown to be present in the EtOH extract of *Map*, but it was not found to be immunogenic [9], despite conflicting reports that suggest it elicits ovine [10] and bovine antibodies [7]. The lipopeptide IIß, isolated from the *Map* EtOH extract, does exhibit a strong antibody response in *Map*-infected cattle [9]. In this study, biochemical and molecular approaches were taken to better define this complex, but useful, EtOH extracted antigen. 

## 2. Materials and Methods 

### 2.1. Antigen Preparation 

The EtOH extract preparations from *Map* K-10 (bovine isolate), *Map* Linda (human isolate), *M. bovis* (HC2005T), *M. avium* (TMC706 and TMC721) and other mycobacteria were produced by gentle vortex in 80% EtOH and centrifugation as described previously [4]. Briefly, *Map* and other mycobacteria were harvested from liquid Middlebrook 7H9 cultures at stationary phase and centrifuged at 2600× *g* for 10 min; the pellet was resuspended in 80% EtOH, agitated by vortex at room temperature for 2 min, and centrifuged at 10,000× *g* for 10 min. EtOH supernatants were dried, resuspended in 1.0 mL of dH_2_O, sonicated briefly to hasten dispersion, aliquoted and frozen. Preps were started with 500 mg to 1 g wet weight of bacteria which yielded 40 to 100 mg of dried material. In the SDS-PAGE experiment, the *Map* EtOH extract was treated with the indicated volume of proteinase K (20 mg/mL; Qiagen, Germantown, MD, USA) for 2 h at 50 °C using the volumes indicated in the results. In the ELISA experiment, to measure antibody binding, proteinase K (200 µg/mL; ACROS Organics-Thermo Fisher Scientific, Pittsburgh, PA, USA) was used.

### 2.2. Antibodies

Monoclonal antibodies (mAb) to *Map* proteins were obtained and characterized as described previously [11]. Briefly, mice were immunized with a whole-cell sonicated extract of *Map*, and stable hybridomas were obtained and corresponding antigens to monoclonal antibodies secreted from these hybridomas were characterized by various means [11,12]. The *M. bovis* MPB83 monoclonal antibody, 1F11, was identified from hybridomas of mice immunized with a sonicated extract of *M. bovis*, as described previously [13].

Rabbit polyclonal antibodies were prepared against the *Map* K-10 EtOH extract in two New Zealand white rabbits (3993 and 3995) using a standardized regimen as described previously [14]. All antibodies used in this study, along with their characteristics, are listed in Table 1.

### 2.3. Folch Lipid Extraction, Thin Layer Chromatography, and Lectin Binding Assays

1 mL of the 80% EtOH extracted antigen prep was dried and further purified using a variation of the Folch extraction method, resuspended in 1 mL of CHCl_3_/MeOH (2:1), and rocked for 2 h. The Folch extraction was performed by adding 0.2 volumes of water, rocking for 1 h, and centrifuging at 10,000× *g* for 10 min. The CHCl_3_ layer was concentrated by drying overnight in a fume hood. For one-dimensional thin layer chromatography (TLC), the chloroform fraction was dissolved in chloroform at the concentration of 50 µg/mL and 10 µL of the solution was loaded onto aluminum-backed Silica Gel 60 plates (Merck) and then developed with CHCl_3_/MeOH/H_2_O (80:10:1). Developed plates were sprayed to detect general components with Ce(SO_4_)_2_/(NH_4_)_2_MoO_4_ in 2 M sulfuric acid and charred.

*Map* was extracted with 80% EtOH and fractionated using the Folch method into organic, interface, and aqueous fractions. All of the fractions were dissolved in methanol at the concentration of 10 µg/mL and 100 µL of each fraction was aliquoted into a 96-well plastic microtiter plate. After evaporating methanol for immobilization of the lipids (and other molecules) onto the wells, the wells were incubated with serum samples (1:100 dilution) collected from JD-positive and negative cattle. Antibody binding was detected by using peroxidase-conjugated secondary antibody (1:1000 dilution) and peroxidase substrate (ABTS) and obtaining quadruplicate measurements at absorbance of 415 nm. The presence of protein in each fraction was confirmed by the ninhydrin test [15].

For the lectin binding assay, all fractions were again dissolved in methanol at a concentration of 10 µg/mL and 100 µL of each fraction was added per well to a 96-well microtiter plate. After evaporating methanol for immobilization of the extract onto the microtiter plate, the wells were incubated with one of four lectins: concanavalin A (ConA), wheat germ agglutinin (WGA), Solanum tuberosum lectin (STL), Limulus Polyphemus lectin (LPL), each at 100 µg/mL with biotin conjugation. Lectin binding was detected by using peroxidase-conjugated NeutrAvidin (1:500 dilution; ThermoFisher, Waltham, MA, USA) and peroxidase substrate (ABTS). Washing was performed with 10 mM phosphate buffered saline (PBS) containing 0.05% Tween 20 (PBS-T) and PBS-T containing 1% bovine serum albumin (BSA, gamma globulin-free; Sigma, St Loius, MO, USA) was used for blocking and dilution of lectin and avidin. Controls with either no bacteria or no lectin were used to measure nonspecific binding of the lectin or NeutrAvidin conjugate. Quadruplicate measurements were taken at an absorbance of 450 nm.

### 2.4. Flow Cytometry Analysis 

Mycobacterial species were cultured in Middlebrooks 7H9 medium supplemented with ADC enrichment (for *M. kansasii* and *M. phlei*) or OADC enrichment (for *M. avium* strains) or OADC and mycobactin J (2 mg/L; for *Map* strains). Bacterial cells were harvested by centrifugation and washed in PBS (pH 7.0) with 10% Superblock (Thermofisher, Waltham, MA, USA) and 0.05% tween 80 (Sigma). The pellet, containing approximately 1 × 10^8^ mycobacteria, was suspended in 100 µL of Superblock buffer containing 2 µL of a test or control serum sample, incubated at room temperature for 1 h, centrifuged at 2500× *g* for 10 min, washed twice with Superblock buffer, resuspended in 50 µL of Superblock buffer containing 1 µL of fluorescein isothiocyanate (FITC)-labeled rabbit α-bovine IgG antibody (heavy and light chains) and incubated at room temperature for 1 h. Bacteria were then suspended in 1 mL PBS (pH 7.0) and subjected to flow cytometry analysis (LSR II, BD Bioscience, San Diego, CA, USA). Bovine antibodies were labeled with FITC and rabbit antibodies were labeled with phycoerythrin (PE). All experiments were performed in triplicate.

### 2.5. ELISA to Measure Antibody Binding 

The *Map* ethanol extract (4 mg) was dried under a fume hood at room temperature overnight.

In the protease experiment, 10 µL of trypsin (Thermofisher, final concentration 200 µg/mL) or proteinase K (ACROS, final concentration 200 µg/mL) was added along with 40 µL of 50 mM Tris buffer (pH 8.0)-20 mM CaCl_2_. These protease reactions were incubated at 37 °C for 3 h and then diluted 1:10 with EtOH. The protease treated and control extracts were added in 50 µL aliquots into each well of a 96-well plate (PolySorpTM Nunc-Immuno 96 microwell plate). For the ConA-agarose experiment, agarose beads covalently attached to ConA were obtained from Vector Laboratories (Burlingame, CA, USA). Both ConA-agarose and agarose were washed in 2 mM CaCl_2_ and briefly centrifuges to collect the beads. The same wash was repeated three times and then 20, 40 and 80 µL of a 50% suspension of beads were added to 10 µg *Map* EtOH extract. The reaction volume was increased to 400 µL with 2 mM CaCl_2_ and allowed to incubated at room temperature for 2 h with rotation. Then the reaction mixtures were centrifuged briefly to remove beads and the supernatant was advanced to the ELISA assay. To conduct the ELISA tests, the plates were stored in a fume hood at room temperature overnight to dry and immobilize ethanol-extracted materials. Each well was treated for non-specific protein binding with 200 µL of Superblock buffer at room temperature for 1 h and then the wells were washed twice with 100 µL of PBS-T. The primary bovine antibody from fecal positive animals was diluted to 1:100 in Superblock buffer and incubated at room temperature for 1 h. The wells were washed four times with 100 µL of PBS-T. For the secondary antibody, each well had 50 µL of α-bovine IgG-HRP conjugate (Jackson ImmunoResearch, West Grove, PA, USA) diluted 1:500 with Superblock buffer added at room temperature for 1 h. The wells were again wash four times with PBS-T. Then 100 µL of a solution of ABTS (1,2’-Azinobis [-ethylbenzothiazoline-6-sulfonic acid]-diammonium salt; Thermo Scientific PIERCE, Waltham, MA, USA) was added to each well. The ABTS solution was freshly prepared using a tablet dissolved in 10 mL of 0.1 M citrate-phospate buffer (pH 5.0) containing 10 µL of 30% hydrogen peroxide. Optical density of the reaction mixture was measured at 415 nm at 2-min intervals for a total of 12 min using a microplate reader (Model 680, BioRad, Richmond, CA, USA).

### 2.6. Construction and Screening of a Map Genomic Expression Library 

A genomic expression library was previously constructed in the lambda ZAP phage by cloning 3–5 kb segments of *Map* ATCC 19698 genomic DNA. The phage expression library was used in this study, but the details of its construction are described previously [14]. Phage harboring cloned DNA inserts of *Map* were plated on lawns of *E. coli* XL-1-Blue MRF’ produced on NZY agar plates (ThermoFisher Scientific, Waltham, MA, USA). Plaques that developed overnight were transferred to 0.01 M IPTG-coated nitrocellulose filters (Protran BA85, Whatman GmbH, Dassel, Germany). The nitrocellulose plaque lifts from each plate were blocked in PBS-tween 20 (0.05%) with 2% BSA (PBS-BSA) for at least 1 h before immunoscreening plaques. They were next probed with hyperimmune serum from rabbits immunized with the EtOH extract of *Map*. These sera were pre-adsorbed with an equal volume of *E. coli* extract (Promega, Madison, WI, USA) at 20 mg/mL for 1.5 h at room temperature with occasional mixing. The pre-adsorbed rabbit serum was diluted 1:400 in blocking buffer. The nitrocellulose filters were exposed to the primary antibody for 2 h followed by 3 washes in PBS-T. Blots were incubated for 1.5 h in protein A-peroxidase (ThermoFisher Scientific, USA) diluted 1:20,000 in PBS-BSA. The blots were again washed three times as described above and developed for chemiluminesence with Supersignal West Pico PLUS reagent (ThermoFisher Scientific, USA). Plaques that reacted with the serum were selected for further study by picking and plaque purification. 

### 2.7. Electron Microscopy 

*Map* cells cultured in Middlebrooks 7H9 and washed in 1x PBS were embedded in epoxy resin and used from a previous study [16]. Fixation and staining procedures were conducted at room temperature. Thin sections for immunoelectron microscopy were washed in 0.1 M cacodylate buffer, pH 7.4 three times for 15 min each and etched with saturated sodium metaperiodate for 15 min. Cells were then blocked with 5% BSA for 30 min. Cells were treated with affinity purified rabbit IgG against *Map* (diluted 1:200) in the blocking solution for 2 h at room temperature. Cells were washed in Tris buffer containing 0.1% Tween 20 and 0.1% BSA four times for 10 min each and then incubated with goat α-rabbit IgG conjugated to colloidal gold (10 nm diameter) in Tris buffer for 2 h. Immunolabeled sections were washed in Tris buffer four times and fixed with 1% glutaraldehyde in Tris for 10 min. All ultrathin sections were double stained with uranyl acetate and Reynolds lead citrate and then observed under a Philips microscope. 

For scanning electron microscopy, mycobacteria (1-mL of confluent culture containing approximately 20 µL of packed bacilli) cultured in Middlebrook’s medium were harvested by centrifugation at 3500× *g* for 10 min, and the pellet washed with buffer A consisting of PBS (pH 7.4) containing 10% Superblock (PIERCE Thermofisher) and 0.05% Tween 80 (ACROS Organics, Morris Plains, NJ, USA). 20 µL of the pellet were resuspended in 100 µl buffer A containing 2 µL of JD-positive serum, JD-negative serum, fetal bovine serum (negative control), or no serum sample, incubated at room temperature for 1 h, centrifuged at 2500× *g* for 10 min, washed twice with buffer A, resuspended in 100 µL buffer A containing 20 µL of colloidal gold particle (20 nm)-labeled rabbit α-bovine IgG antibody (EY Laboratories, INC., California, CA, USA) and incubated at room temperature for 1 h [1]. The ethanol extraction as described in antigen extraction was performed on a subset of labeled bacteria. The bacteria were then washed in 200 µL PBS (pH 7.4), and centrifuged at 3500× *g* for 10 min. This step was repeated once using Hank’s balanced salt solution (HBSS). Bacterial pellets were resuspended in 100 µL HBSS and placed in a well containing a Silicon Chip (Ted Pella INC., Redding, CA, USA) and then incubated at room temperature for 30 min before gently decanting liquid. Two-hundred µl of primary fixative containing 2% paraformaldehyde, 2.5% glutaraldehyde, 0.1 M sodium cacodylate buffer (pH 7.4), and 0.2 M sucrose, was added to the well and incubated for at least one hour at room temperature to overnight at 4 °C. Primary fixative was gently decanted and 200 µL secondary fixative containing 2% OSO_4_ and 0.1 M sodium cacodylate buffer (pH 7.4) was added and incubated for 1–2 h at room temperature. A series of sequential ethanol washes were performed for at least 10 min each in 50% 70%, 95%, and 100% EtOH before drying samples under CO_2_ using a critical point drier apparatus [17] before drying samples under CO_2_ using a Ladd critical point dryer (Ladd Research, Williston, VT, USA). Samples were immediately examined in a Leo 1525 scanning electron microscope (Carl Zeiss SMT, Inc., Thornwood, NY, USA). Slides of cultures were prepared in duplicate and no fewer than 30 cells were observed from each sample. 

### 2.8. Statistical Analysis 

Probabilities were conducted to determine if the null hypothesis could be rejected. *p* values were calculated by *T*-test in Microsoft Excel and GraphPad Prism version 8.1.2. *p* values of less than 0.01 enabled rejection of the null hypothesis.

## 3. Results

### 3.1. Bovine and Rabbit Antibodies React to the Surface of Map Bacteria

**Figure 1:**
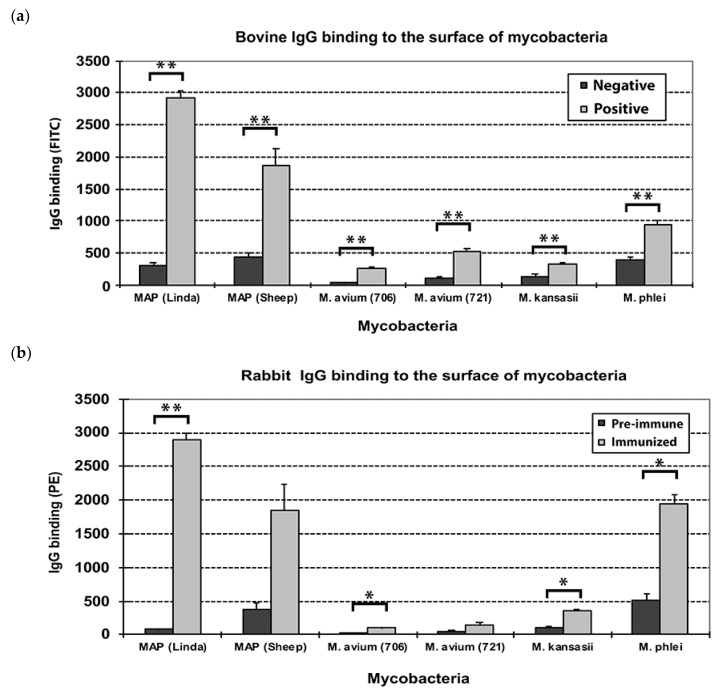
Antibody binding to intact mycobacteria with bovine and rabbit antisera. (**a**) Shown are the results from 5 Johne’s disease (JD)-positive (gray bars) and 5 JD-negative cows (black bars) against different species and subspecies of mycobacteria. (**b**) Sera from 2 rabbits immunized with the *Map* K-10 EtOH extract shows a similar pattern to what was observed with sera from cattle with JD. The pre-immune serum is represented by black histogram bars and post-immune serum is the gray bars. Error bars represent standard deviation of the means. *M. avium* (706) is TMC706 and *M. avium* (721) is TMC721, a strain isolated from a child’s lymph node and is serotype 3. Significance is denoted by asterisks, * = *p* < 0.01; * * = *p* < 0.001.

*Map* and other mycobacteria were treated with sera from JD-negative cows, JD-positive cows, pre-immune and EtOH-extract immunized rabbits. Bound IgG molecules on the surface of the bacteria were labeled with FITC-(for bovine IgG) or PE-(for rabbit IgG) conjugated secondary antibody and then analyzed by flow cytometry similar to what has been done previously with intact *Map* [1]. Both the bovine (Figure 1a) and rabbit sera (Figure 1b) displayed similar results, with the highest binding to the Linda strain in each. This result demonstrates that the bovine and rabbit serum contain *Map*-specific IgG, and the human strain (Linda) showed higher levels of antibody binding than did the sheep strain. Some background reactivity was noted with *M. phlei*, especially with the rabbit sera. This mycobacterial strain is typically used as a pre-absorbent in ELISA tests for JD [18]. Very little cross reactivity was associated with the two *M. avium* subspecies *avium* strains and *M. kanasii* (Figure 1).

**Figure 2:**
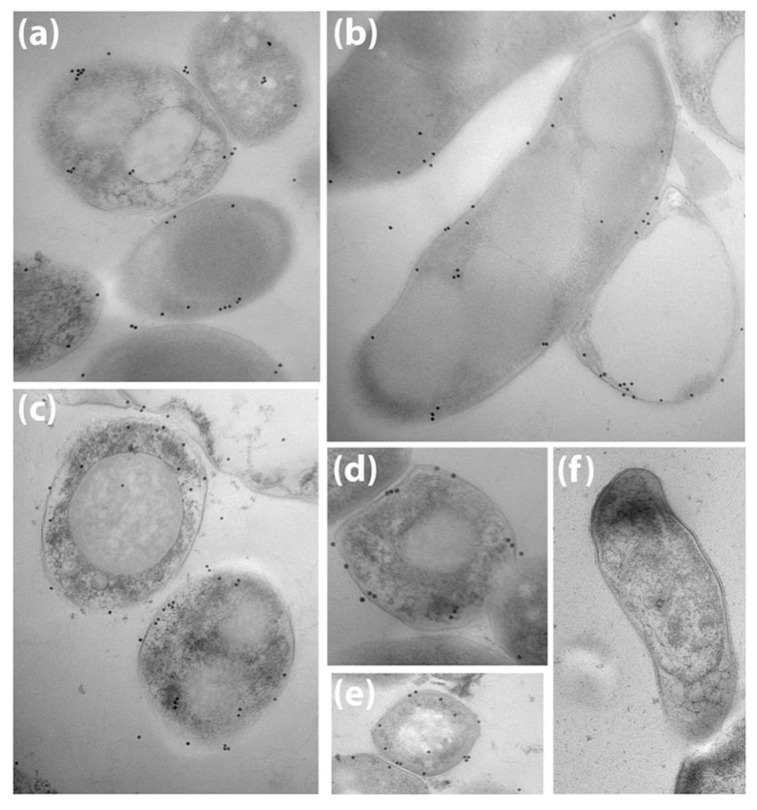
Transmission electron microscopy of *Map* K-10 exposed to rabbit pre-immune and post-immune serum. Detection of antibody binding was with Colloidal gold 10 nm particles. The images are *Map* strain K-10 labeled with antibody against the EtOH prep from rabbit 3993 (**a**–**c**) and 3995 (**d**,**e**). Pre-immune serum from 3993 does not react to *Map* (**f**). Images are all magnified 49,000×.

**Figure 3:**
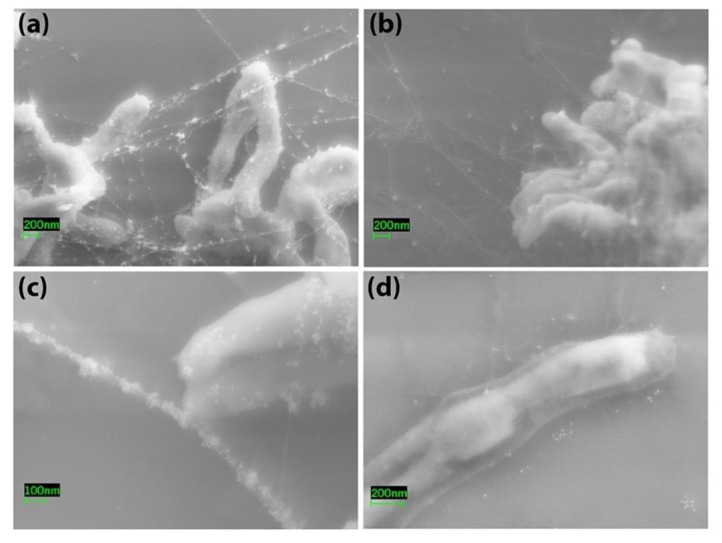
Scanning electron microscopy of *Map* pre and post EtOH extraction. *Map* were exposed to bovine serum from a JD-positive (**a**,**c**,**d**) and a JD-negative cow (**b**). The images in (**a**–**c**) were taken prior to EtOH extraction while (**d**) was taken after EtOH extraction. Note the clear zone at the surface indicating the stripped nature of the cell wall in (**d**). Magnification is indicated by size bars in the lower left corner of each image.

*Map* was also examined by immunoelectron microscopy using rabbit anti-sera raised against the *Map* EtOH extract. Antibodies bound on the surface of the bacteria as shown by transmission electron microscopy using colloidal gold particles to indicate the location of antibody binding (Figure 2a–e). No antibody binding was evident with pre-immune serum (Figure 2f). In addition, immune complexes appearing as “pearls on a string” by scanning electron microscopy were prevalent when *Map* was treated with serum from JD cows (Figure 3a,c) and less so with negative serum (Figure 3b). *Map* treated after 80% EtOH extraction showed a clear, translucent zone around the bacilli indicating the stripped nature of the bacilli surface, which suggested that the outer layer of the bacteria has been removed (Figure 3d). Finally, when EtOH extracted cells were exposed to JD-positive cow serum, antibody binding levels appeared lower and comparable to that observed with negative serum on pre-extracted cells (Figure 3d). 

Collectively, these experiments demonstrated antibody reactivity at the bacilli surface to component(s) in the EtOH extract, but it is not clear if the reactivity is to protein, carbohydrate or lipid molecules. A unique look at the *Map* bacilli post-EtOH extraction was also observed by scanning electron microscopy. Next, we examined the composition of these extracts.

### 3.2. EtOH Extracts of Mycobacterial Species Show Markedly Different TLC Profiles

**Figure 4:**
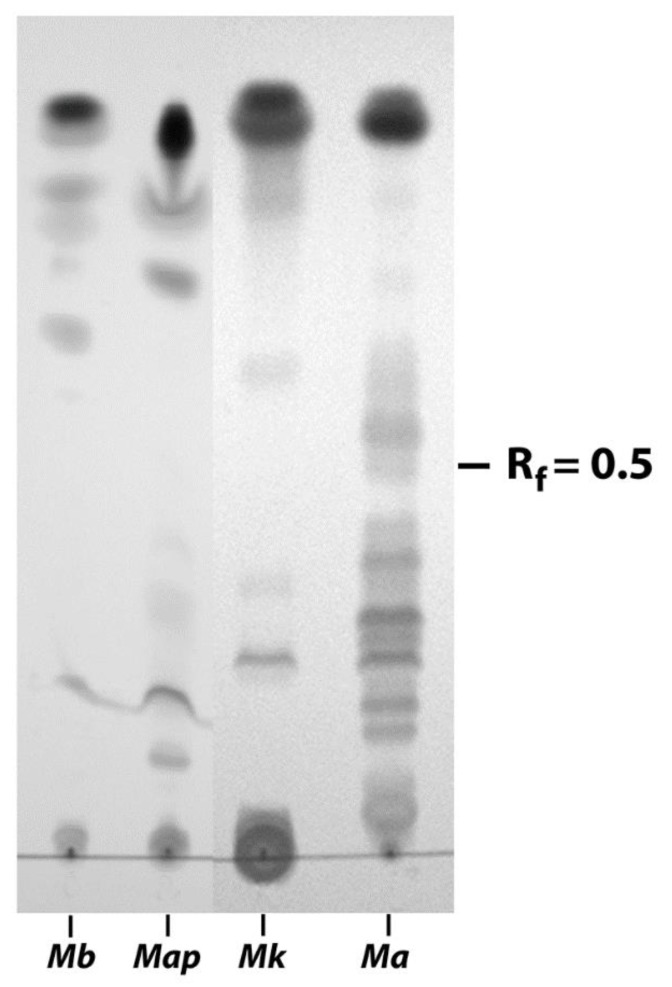
Thin layer chromatography of mycobacterial lipids. Shown are the lipid migration patterns of *M. bovis* (Mb), *Map* K-10 (Map), *M. kansasii* (Mk) and *M. avium* subspecies avium (Ma). Plates were developed in chloroform-methanol-water (90:10:1) until this solvent phase migrated 14 cm. The R_f_ = 0.5 indicates the solvent migration halfway point. R_f_ symbolizes retardation factor.

Because the EVELISA is better than a sonicated whole-cell extract ELISA at distinguishing infected cattle from healthy cattle [3], we sought to define some of the components of this antigen prep. The EtOH extracts from different mycobacterial strains were analyzed by TLC and showed strikingly distinct profiles in their lipid migration patterns. *M. bovis* had most of its lipids migrating far up the silica gel plate resulting in R_f_ values above 0.5. In contrast, *M. avium* subspecies avium showed the majority of lipids migrating below R_f_ values of 0.5 (Figure 4). All four mycobacterial species showed a predominant lipid at the top of the solvent migration line. Importantly, *Map* and *M. avium* could be easily distinguished based on their lipid migration patterns by TLC even though they are genetically highly related subspecies [19]. Researchers have also demonstrated unique lipid profiles between these closely related subspecies using 2-dimensional TLC [9]. This suggests that unique lipids of Map could impart specificity to potential diagnostic tests.

### 3.3. Identification of Proteins, Lipids and Carbohydrates in the EtOH Extract

**Figure 5:**
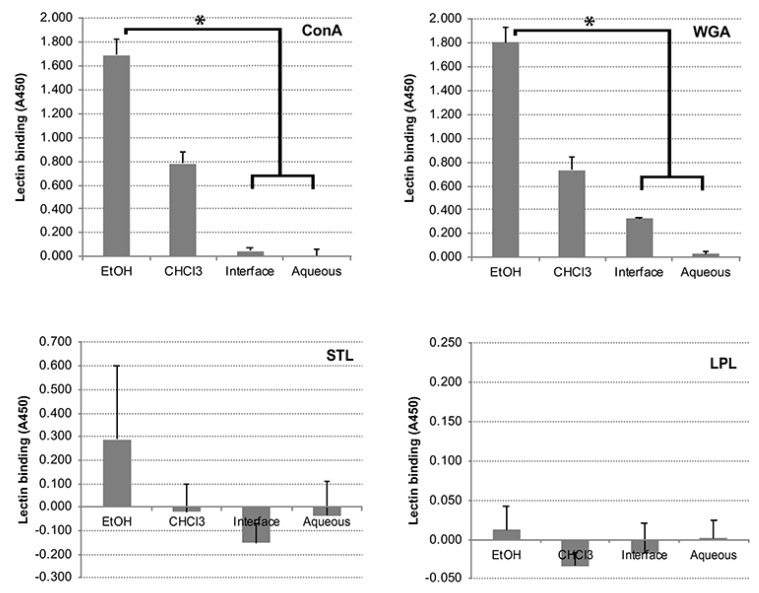
Lectin binding to the *Map* EtOH extract. Lectin binding was detected using peroxidase-conjugated secondary antibody and peroxidase substrate (ABTS) followed by measuring absorbance at 415 nm. Histogram bars represent mean lectin binding ± standard deviation of quadruplicate determinations. Binding to both concanavalin A (ConA) (top left) and wheat germ agglutinin (WGA) (top right) was observed predominantly in the chloroform phase with little to no binding in the aqueous phase. Conversely, no Solanum tuberosum lectin (STL) (bottom left) or Limulus Polyphemus lectin (LPL) (bottom right) binding was observed in any fraction of the extract, while a low but detectable binding level was observed in the complete extract with STL. An asterisk denotes *p* values less than 0.01.

The presence of glycoproteins in the EtOH extract was determined using three well-known plant lectins and one vertebrate lectin. ConA is an α-D-mannose/α-D-glucose-binding lectin that recognizes N-glycans and is not known to bind common O-glycans on glycoproteins [20], whereas wheat germ agglutinin (WGA) binds to the simple sugar N-acetyl-D-glucosamine [21]. Both lectins reacted strongly to the EtOH extract indicated the presence of both types of glycoproteins (Figure 5). Binding of ConA and WGA was reduced in the chloroform and interface fractions while minimal in the aqueous fraction (Figure 5). In contrast, the LPL and STL lectins, which bind to N-acetylneuraminic acid and oligomers of N-acetylglucosamine, respectively, showed little or no binding to the *Map* EtOH extract (Figure 5). This lack of binding suggests those carbohydrates were either not present in the *Map* extract or are not available for binding.

Although the complete, unfractionated EtOH extract showed the strongest lectin binding, the chloroform fraction displayed the most binding among the fractions. There were several glycolipids present in the cell wall of *Map* that could account for lectin binding, most notably, LAM (a mannose-rich compound) and peptidoglycan which contains N-acetylglucosamine.

**Figure 6:**
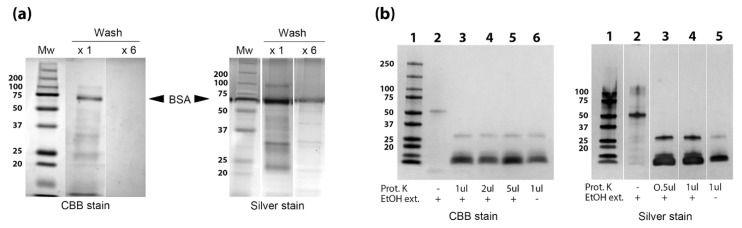
Preparation of EtOH extract for mass spectrometry analysis. (**a**) *Map* bacilli were washed 6 times prior to EtOH extraction (labeled × 6) to avoid bovine serum albumin (BSA) contamination. A second extract was prepared after washing *Map* once (label × 1). Lipids and carbohydrate were removed from the EtOH extract through a second chloroform:methanol:water extraction. This second extraction was run on 12% SDS-PAGE denaturing gels and exposed to Coomassie stain (CBB) and silver stain. The location of BSA migration is identified for both gel images. (**b**) Proteinase K treatment of *Map* EtOH extract. Inclusion of proteinase K or the EtOH extract along with staining method is indicated beneath the gel. Kilodalton size markers are shown in the left margins of all gels.

To isolate proteins away from the abundant lipid and carbohydrate components present in the EtOH extracts, the *Map* bacteria were first washed and then subjected to EtOH extraction and further extracted with chloroform:methanol:water (2:1:0.6). The methanol fraction, containing the proteins, was collected and analyzed by SDS-PAGE stained with Coomassie and silver nitrate (Figure 6a). Proteinase K digestion was conducted to confirm that the methanol fraction contained proteins (Figure 6b). However, when tandem mass spectrometry (LC-MS/MS) was used to assay the methanol extracted proteins from the EtOH prep, only albumin and trypsin were detected in these experiments. This result was consistent even when bacteria were washed 6 times, which appeared to remove most of the BSA. This extensive washing regimen also prevented recovery of the *Map* proteins which could only be detected by silver staining, not by Coomassie stain (Figure 6a).

Therefore, we took a more classical approach and analyzed EtOH-vortex preps with a panel of monoclonal antibodies to investigate mycobacterial proteins and determine their presence or absence.

A total of 17 mAbs against known proteins were tested, but only MAP_2698c and LAM were present in the *Map* EtOH extract (Table 2). MAP_2698c or *desA2* encodes a fatty acid desaturase which is involved in fatty acid metabolism important for mycobacterial cell wall maintenance. This protein has shown promise as a subunit vaccine because it produces strong IFN-γ responses in vaccinated sheep [22]. Surprisingly, the 35 kDa major membrane protein was not present in this extract, as confirmed by the lack of reactivity with two mAbs to independent epitopes within this protein [23]. 

**Figure 7:**
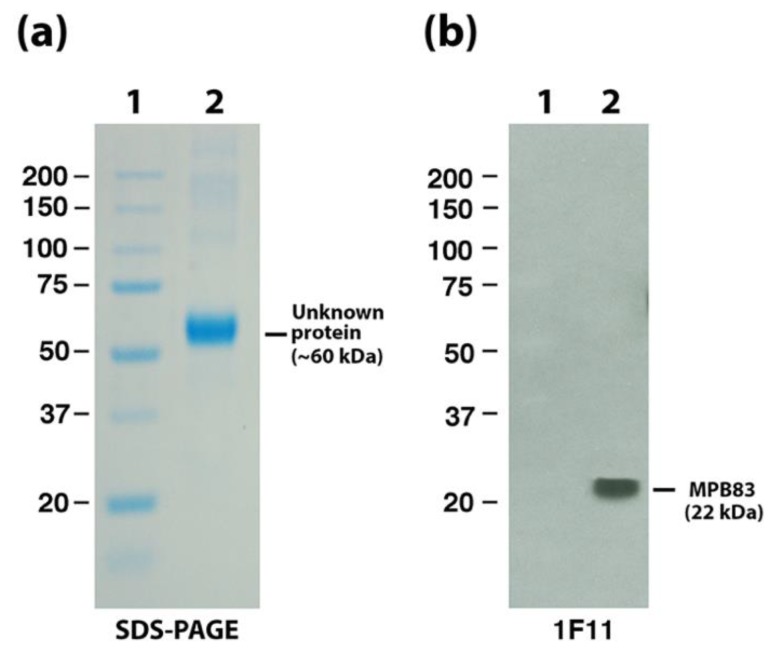
MPB83 is not the most abundant protein in *M. bovis* EtOH extracts. (**a**) SDS-PAGE analysis of the *M. bovis* EtOH extract reveals a high abundant protein migrating between the 50 and 75 kDa size markers (lane 2). (**b**) Immunoblot analysis with a monoclonal antibody to MPB83 (1F11) identifies the 22-kDa MPB83 protein in the EtOH extract (lane 2). Protein size markers are loaded in lane 1 with sizes identified in kilodaltons (kDa) in the left margins. The unknown and MPB83 proteins are indicated in the right margins.

The MPB83 protein from *M. bovis* has long been reported as a surface-exposed lipoglycoprotein in *M. bovis* [24,25]; this protein is present in EtOH extracts, as demonstrated previously [4]. MPB83 was confirmed in *M. bovis* EtOH extracts at the expected size of 22-kDa in this study (Figure 7b), although it does not correspond to the most abundant protein present in these extracts. That protein is unknown and migrates between 50 and 75-kDa standards in stained SDS-PAGE gels (Figure 7a). The *Map* EtOH extract also has a similar sized abundant protein (Figure 6b, lane 2).

### 3.4. Identification of MAP_0585 in a Map Expression Library

**Figure 8:**
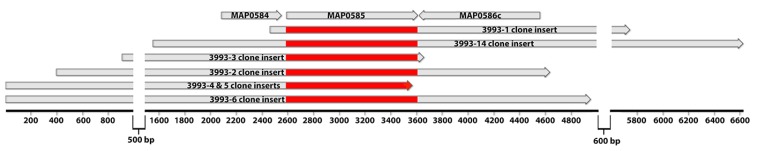
Sequence alignment of positive clone inserts from a *Map* phage expression library. Shown are the DNA inserts subcloned from positive plaques obtained from the library screen. Subcloned inserts are aligned and drawn relative to a base pair scale that spans 6.6 kb. There is a 500 and 600 bp break in the scale to amplify the region of interest. The top row shows the coding sequences from the *Map* genome and their relative positions among the library clones. Highlighted in red is the region of the respective clones that overlap with the coding sequence MAP_0585. Only this coding sequence was universally present in all the clones.

A *Map*-lambda expression library was previously constructed [3] and used in this study to identify additional proteins present in the EtOH extract. Approximately 950,000 plaques from this library were screened with rabbit hyperimmune sera raised against the *Map* EtOH extract. Seven positive phage plaques were identified from this screen. However, when these plaques were subcloned into the pBK-CMV plasmid by in vivo excision, one of the plaques lost reactivity leaving six positive clones (Figure S1). All clones revealed a similar sized protein by Western blot suggesting these clones may be expressing the same antigen (Figure S1). Sequence analysis and alignment of the clone inserts shows that MAP_0585 is the only coding sequence common in all clones (Figure 8). This is a hypothetical protein that has conserved domains suggestive of nucleic acid binding. 

**Figure 9:**
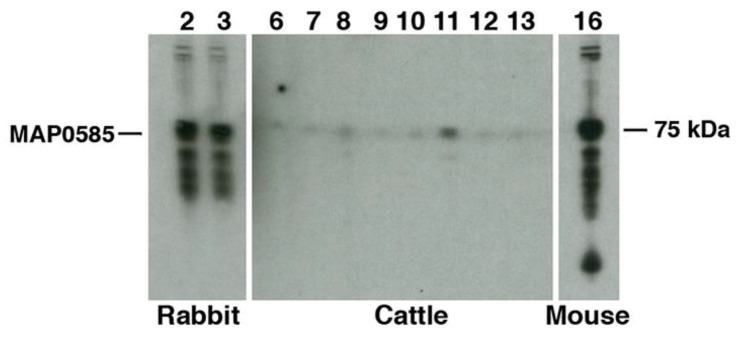
Antigenicity of MAP_0585. Immunoblot analysis shows MAP_0585 reacts strongly to the rabbit antisera developed to the EtOH extract (3993 in lane 2 and 3995 in lane 3) and the protein is also detected by a mAb to the maltose bind protein affinity tag (lane 16). However, the protein is not antigenic in cattle (lanes 9 and 12 are healthy cattle and all other lanes are JD cattle). The position of the MAP_0585 is indicated in the left margin and the host generating antibody is indicated beneath the blot.

The full-length recombinant proteins of MAP_0585 and MAP_2698c were previously expressed and purified from *E. coli* [26]. MAP_0585 showed an unusual absorbance peak at 270 nm, which has been observed with only one other *Map* protein (MAP_0790) from a collection of over 800 such proteins expressed in *E. coli* [26]. Attempts to produce mAbs to MAP_0585 were unsuccessful, as no positive hybridomas were obtained after immunizing mice. However, MAP_0585 does react strongly to the rabbit hyperimmune sera (Figure 9).

### 3.5. Carbohydrates, but Not Proteins in the EtOH Extract Are Immunogenic in Cattle

The two *Map* recombinant proteins identified in the EtOH extracts were tested previously for the ability to discriminate JD cows from healthy animals using protein arrays [27]. MAP_2698c was not immunogenic in those studies and MAP_0585 showed some promise as an antigen since it ranked in the upper half of all proteins examined. To examine this potential antigen further, a Western blot of MAP_0585 showed strong reactivity with the α-EtOH extract rabbit sera and the α-MBP mAb, both of which highlighted the 75 kDa fusion protein representing MAP_0585. However, results show a general lack of reactivity with JD cattle (Figure 9), while little to no background reactivity was also observed with the negative cows.

**Figure 10:**
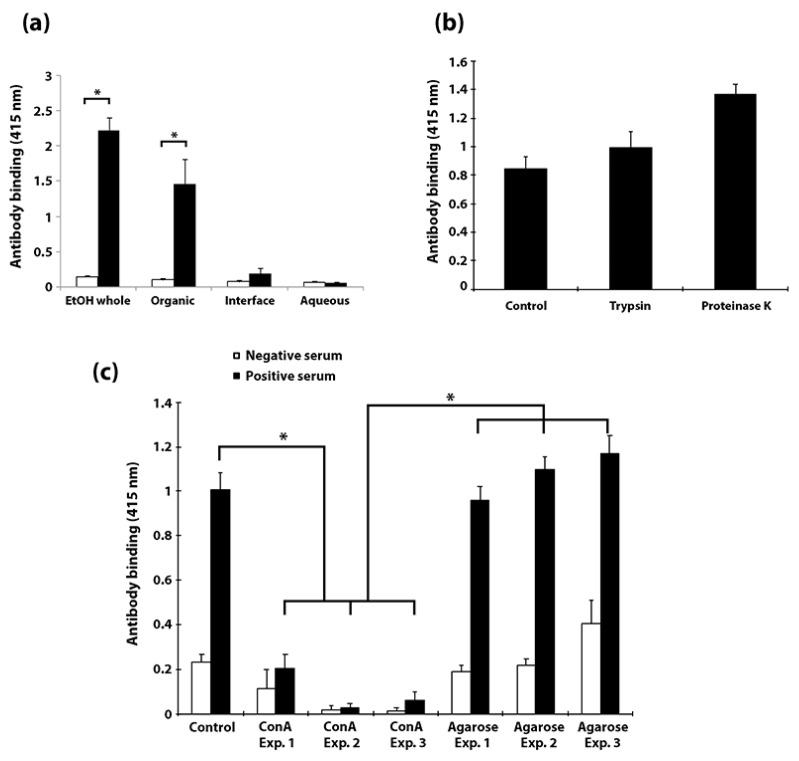
Bovine antibody binds to a carbohydrate component of the *Map* EtOH extract, not to protein. (**a**) Surface antigens of *Map* K-10 were extracted with 80% ethanol (EtOH Whole) and fractionated by Folch extraction method into organic (chloroform), interface and aqueous fractions. After evaporating methanol for immobilization of the lipids (and other molecules) onto the wells of a microtitre plate, they were incubated with serum samples (1:100 dilution) collected from JD-positive (solid bar) and negative (open bar) cattle. Histogram bars represent mean antibody binding ± standard deviation of quadruplicate determinations. Most of the antigenicity is in the organic fraction. (**b**) EtOH extract treated with proteases shows no negative effects on bovine antibody binding. The extract was treated with either 10 µg/mL trypsin or 10 µg/mL proteinase K with each treatment actually enhancing antibody binding. This enhancement was not statistically significant. However, EtOH extract antigens are efficiently removed by ConA-agarose as shown by lack of antibody binding (**c**). Absorption with agarose only does not affect antibody binding to the EtOH extract. This experiment was repeated in triplicate with qraduplicate measurements for each. *p* values less than 0.01 are denoted by an asterisk.

To obtain a broader view of the antigenicity of all proteins present in the extract, EtOH extracted *Map* components were further fractionated by the Folch wash method and acetone precipitated into three fractions: aqueous, interface, and chloroform. Each fraction showed reactivity to antibodies in sera of *Map*-infected cattle; however, antibody binding of the fractions was lower than the complete extract used in the EVELISA test (Figure 10a). This indicated that the complete repertoire of antigens in the EtOH extract is required to maintain the high sensitivity of the EVELISA. Notably, when the EtOH extract was treated with proteinase K, antibody binding did not diminish and in fact was slightly enhanced (Figure 10b). Although additional efforts to define the components in the EtOH extract preparation are needed to better understand which components are important for diagnostics, our data suggest the preparation contains predominantly carbohydrate and lipid components with a small protein component that is not strongly antigenic since most antigenic molecules were fractionated into the organic layer while the aqueous layer, containing proteins, did not bind antibodies (Figure 10a), and protease treatment did not diminish antigenicity (Figure 10b).

## 4. Discussion

In this study, we examined the lipid, polysaccharide, and protein components of the EtOH-vortex extracts of mycobacteria, focusing on *Map* and less so on other mycobacteria including *M. bovis*. Lipids of mycobacterial strains examined showed divergent migration patterns by TLC. The cell wall polysaccharide, LAM, was shown to be present in both EtOH extracts of *Map* and *M. bovis*. The two proteins in *Map* EtOH extracts included a hypothetical protein (MAP_0585) and a fatty acid desaturase (MAP_2698c). These same proteins were either not present in *M. bovis*, or their orthologs are sufficiently different such that the antibodies, produced against *Map* proteins, did not bind. In fact, none of the mAbs, excepting those binding LAM, were detected in the *M. bovis* EtOH extracts.

Due to the method used to prepare the EtOH extract, surface components were expected to predominate. The 80% EtOH and gentle vortex mixing of intact mycobacteria should release only the surface components yet keep the bacilli intact. The bacteria may still be viable since only a brief time is spent in EtOH during the extraction, but viability studies were not conducted as the bacteria were discarded after extraction. However, the efficient removal of the outer surface of these bacteria, as shown in Figure 3d, suggests some interesting future studies could be performed. Once their viability post-extraction is determined, it would be expected that the bacteria show a reduced clumping, biofilm formation, and ability to invade epithelial cells lining the bovine intestine. Also, an increased ability to be phagocytosed by macrophages could be anticipated based on previous studies with the *M. tuberculosis* glycan-rich outer layer which was removed by sonication [28]. Another interesting study would be to determine if the EtOH-extracted bacteria could regenerate their cell walls after one division.

Seven independent library clones were obtained from the expression library screen, and these clones overlapped with each other to produce a 6.6 kb contiguous segment of the *Map* genome, but only the MAP_0585 coding sequence was present among all the clones. This gives a high level of confidence in the screening method and subsequent immunoblot analysis demonstrated that MAP_0585 is strongly reactive with the sera used to screen the library (Figure 9). Additional proteins were not identified despite extensive screening. This may be due to either an under represented library or there could simply be a lack of protein diversity in this extract as discussed below.

The extracts analyzed using SDS-PAGE gel prior to proteinase K treatment showed a number of defined bands that were only detectable by silver stain. Yet even with this sensitive stain, only ten distinct bands are evident. The major membrane protein was not among these. This result was unexpected because this protein, encoded by MAP2121c, has been shown to be a surface exposed protein and an invasin [29]. Only one protein, approximately 60 kDa in size, was abundant enough to be detectable by Commassie stain in mycobacterial EtOH extracts. This protein was not identified in this study, but it is proteinase K sensitive and is easily detectable by both Coomassie and silver stain. It is likely that the 60-kDa band in both *Map* and *M. bovis* extracts are the same, although this has yet to be conclusively demonstrated. A more comprehensive approach is needed to identify additional proteins, but MS-MS is difficult to obtain results for reasons that are only speculative.

Antibodies to the EtOH extract reacted to the surface of the mycobacteria as determined by electron microscopy. LAM is composed entirely of lipid and carbohydrate and is linked to the cell membrane by a lipid anchor [30]. While MAP_2698c is in the membrane as determined by reactivity exclusively with membrane-enriched fractions of *Map* using mAbs to this protein [12]. However, MAP_2698c has no internal helices and no signal peptide. Interestingly, PSORTb protein localization software [31] could not make a final localization prediction for either MAP_0585 or MAP_2698c, although the data from PSORTb suggest periplasmic location for MAP_0585 due to the presence of a signal peptide and 1 internal helix. The *E. coli* expressed MAP_0585 was not detected in JD cows, but the *Map*-expressed protein was highly immunogenic in rabbits as indicated by the antibodies that selected all the library clones. Perhaps this protein is glycosylated in the native host, rendering it more immunogenic. MAP_0585 does have consensus glycosylation sites, but the presence of such consensus sequences only indicates the possibility, not the certainty that glycosylation actually occurs.

Regarding the antigenic components of the EtOH extract, data suggests that the immunoreactive component is not protein, but rather a carbohydrate, lipid or a combination of the two. The lack of reactivity from the recombinant proteins with JD cattle combined with the result that protease treatment did not diminish antibody reactivity supports this conclusion. Indeed, even though MAP_0585 is strongly immunogenic in immunized rabbits, this protein is not detected in *Map*-infected cattle. Still, the protein component of this extract may provide important specificity since carbohydrates and lipids are more conserved structures. However, TLC profiles in this study and others has shown unique lipid patterns among mycobacteria, which could also be a factor in diagnostic specificity. Historically, the *Map* LAM has been considered immunogenic [32,33], but has not outperformed sonicated antigen preps for diagnostics [34]. One major immunogenic molecule in this extract is lipopeptide IIβ, which is a de-methylated derivative of Para-LP-01 [9]. 

Dozens of different lectins and other proteins with carbohydrate-binding domains have been characterized. ConA is a mannose/glucose-binding lectin and a well-known T cell mitogen that can activate the immune system, recruit lymphocytes and elicit cytokine production [35]. With strong cytokine induction abilities, ConA has been ideal as a positive control for IFN-γ production in many *Map* immune response studies. For *M. tuberculosis*, ConA binding was used to identify eight glycoproteins including PhoS1, SodC, LpqH, MPT83, and lipoproteins LppN, LppQ, LprI, and glutamine-binding protein GlnH [36]. The LAM structure is mannose rich and hence it likely binds to ConA as well. In contrast, the carbohydrate that strongly binds WGA is N-acetylglucosamine. Mycobacterial peptidoglycan is composed of alternating N-acetylglucosamine and modified muramic acid residues linked in a β(1→4) configuration. WGA preferentially interacts with dimers and trimers of this carbohydrate. WGA can also bind oligosaccharides containing terminal N-acetylglucosamine, a structure that is common to many membrane glycoproteins. Thus, it is not surprising that *Map* EtOH extracts reacted strongly to this lectin and suggests peptidoglycan is present in these extracts. 

## 5. Conclusions

The EtOH extracted antigen used in the promising EVELISA test for JD is useful for antibody-based diagnostics. The content of this antigen was relatively unknown despite its great potential in JD diagnostics. This study has revealed the complex nature of this antigen prep which mirrors the cell wall complexity of *Map* itself.

## Figures and Tables

**Table 1:** Antibodies used in this study.

Name	Type	Antigen ^1^	Description
17A12	Murine mAb IgG1	MAP_1025	Proline rich antigen; RDD family protein
14C5	Murine mAb IgG2a	MAP_1272c	NlpC/P60; peptidoglycan hydrolase
8G6	Murine mAb IgG1	MAP_1272c	NlpC/P60; peptidoglycan hydrolase
9G10	Murine mAb IgG2a	MAP_1643	Isocitrate lyase, AceAb
8G2	Murine mAb IgG1	MAP_2121c	Major membrane protein (MMP)
14G3	Murine mAb IgG2a	unknown	
14D4	Murine mAb IgG3	MAP_2698c	Fatty acid desaturase
6C9	Murine mAb IgG1	MAP_3060c	Electron transfer protein, α-subunit
9H3	Murine mAb IgG2b	MAP_3404	Acetyl-CoA, biotin carboxylase subunit
11G4	Murine mAb IgG1	MAP_3840	DnaK chaperone; Heat shock protein
14G11	Murine mAb IgG1	MAP_3976	Lipoprotein anchoring transpeptidase
7A6	Murine mAb IgG2a	MAP_3936	Molecular chaperone, GroEL2
12C9	Murine mAb IgG1	MAP_4145	Memb. protein, short C-terminal domain
13E1	Murine mAb IgG1	MAP_2121c	Major membrane protein (MMP)
7C8	Murine mAb IgG2a	MAP_3404	Acetyl-CoA, biotin carboxylase subunit
p9270	Human mAb IgG1	LAM ^2^	Cell wall lipopolysaccharide
p9045	Human mAb IgG1	LAM ^2^	Cell wall lipopolysaccharide
3993	Rabbit polyclonal ab	EtOH ^3^ extract	
3995	Rabbit polyclonal ab	EtOH extract	

^1^ The antigen listed binds to the antibody. For mAb 14G3, the antigen is still unknown. ^2^ LAM is lipoarabinomannan. ^3^ EtOH is ethanol. p9270 and p9045 each bind to different epitopes on LAM.

**Table 2:** Proteins present or absent in the *Map* and *M. bovis* EtOH extracts.

Protein^1^	Description	mAb	*Map* K-10	*M. Bovis*
MAP_1025	Proline rich antigen	17A12	-	-
MAP_1272c	NlpC/P60 protein	14C5	-	-
MAP_1272c	NlpC/P60 protein	8G6	-	-
MAP_1643	Isocitrate lyase, AceAb	11F6	-	-
MAP_2121c	Membrane protein	8G2	-	-
MAP_2121c	Membrane protein	13E1	-	-
MAP_2698c	Fatty acid desaturase	14D4	+	-
MAP_3060c	Electron transfer protein	6C9	-	-
MAP_3404	Acetyl-CoA carboxylase	9H3	-	-
MAP_3404	Acetyl-CoA carboxylase	11B8	-	-
MAP_3840	DnaK chaperone	11G4	-	-
MAP_3976	Lipoprotein anchoring transpeptidase	14G11	-	-
MAP_3936	Molecular chaperone GroEL2	7A6	-	-
MAP_4145	Membrane protein	12C9	-	-
LAM	Lipoarabinomannan	p9045	+	+
LAM	Lipoarabinomannan	p9270	+	+
Mb2898	Surface lipoprotein MPB83	11F1	-	+

^1^ Genome locus tag for specific proteins.

## Supplementary Materials

The following are available online at https://www.mdpi.com/2306-7381/6/4/88/s1, Figure S1: IPTG-induced expression of positive phage subclones obtained from the library screen.
