# Supplementary material for: Characterization of Ethanol Extracted Cell Wall Components of Mycobacterium avium Subsp. paratuberculosis
Source: Vet Sci. 2019 Oct 31;6(4):88. doi: 10.3390/vetsci6040088 (PMC6958465; doi:10.3390/vetsci6040088)
Supplement: Supplementary file 1 [file vetsci-06-00088-s001.pdf]

veterinary sciences

Supplementary Materials:

Characterization of Ethanol Extracted Cell Wall Components of *Mycobacterium avium* Subsp. *paratuberculosis*

John P. Bannantine *, Ashutosh Wadhwa, Judith R. Stabel and Shigetoshi Eda

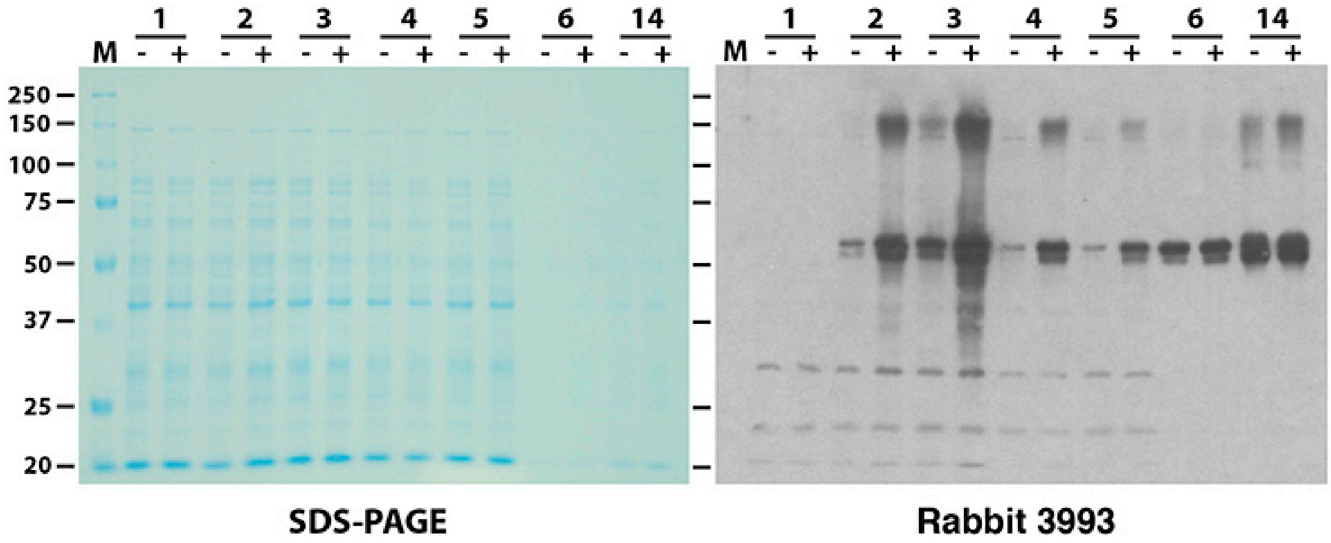

Figure S1. IPTG-induced expression of positive phage subclones obtained from the library screen. Shown is a Coomassie stained SDS_PAGE and corresponding immunoblot of the E. coli clone extracts. Immunoblot was probed with the rabbit 3993 antisera (1:1,000) and shows some of the clones are IPTG inducible while other are constitutive (clone 6 and 14 for example). A similar sized band between the 50 and 75 kDa markers is detected by the antibodies in all clones except clone 1, suggesting it has undergone a deletion or other modification since the subcloning step. Protein size markers are labeled "M" and clone numbers are indicated above their plus or minus symbols, which indicate exposure to IPTG.
